# EEG Resting-State Brain Topological Reorganization as a Function of Age

**DOI:** 10.1155/2016/6243694

**Published:** 2016-02-24

**Authors:** Manuela Petti, Jlenia Toppi, Fabio Babiloni, Febo Cincotti, Donatella Mattia, Laura Astolfi

**Affiliations:** ^1^Department of Computer, Control, and Management Engineering, “Antonio Ruberti” Sapienza University of Rome, Via Ariosto 25, 00185 Rome, Italy; ^2^Neuroelectrical Imaging and BCI Lab, Fondazione Santa Lucia, IRCCS, Via Ardeatina 306, I-00179 Rome, Italy; ^3^Department of Molecular Medicine, Sapienza University of Rome, Viale Regina Elena 291, 00161 Rome, Italy

## Abstract

Resting state connectivity has been increasingly studied to investigate the effects of aging on the brain. A reduced organization in the communication between brain areas was demonstrated by combining a variety of different imaging technologies (fMRI, EEG, and MEG) and graph theory. In this paper, we propose a methodology to get new insights into resting state connectivity and its variations with age, by combining advanced techniques of effective connectivity estimation, graph theoretical approach, and classification by SVM method. We analyzed high density EEG signals recorded at rest from 71 healthy subjects (age: 20–63 years). Weighted and directed connectivity was computed by means of Partial Directed Coherence based on a General Linear Kalman filter approach. To keep the information collected by the estimator, weighted and directed graph indices were extracted from the resulting networks. A relation between brain network properties and age of the subject was found, indicating a tendency of the network to randomly organize increasing with age. This result is also confirmed dividing the whole population into two subgroups according to the age (young and middle-aged adults): significant differences exist in terms of network organization measures. Classification of the subjects by means of such indices returns an accuracy greater than 80%.

## 1. Introduction

In the last years, several anatomical, functional neuroimaging, and electrophysiological studies have investigated the resting-state connectivity in order to understand the effects of aging on the human brain. Functional connectivity studies have suggested that aging is related to decreased connectivity patterns in the Default Mode Network (DMN; [1–4]) but influences also attention networks and sensory-motor systems [5–8]. Also, in EEG field, this phenomenon has been investigated confirming fMRI results [9–14]. Different approaches based on graph theory applied to brain functional networks have been used to describe the effects of aging on cerebral processes. In particular, the resting-state network organization is suggested to tend to a more random configuration by a reduction of overall connectivity (decreased clustering and increased path length): this phenomenon was demonstrated in [10, 11] estimating brain connectivity by means of Synchronization Likelihood and in [14] by means of lagged-phase synchronization. In fact, among indices describing the properties of a brain network, characteristic path length (overall integration), clustering (local segregation), and small-worldness, defined as ratio between them, represent some key topological metrics [15] describing the organization of information flows in a network. On the basis of such metrics, networks can be described as regular, small-world, or random. Several anatomical, functional neuroimaging, and electrophysiological studies demonstrated that brain networks inferred from healthy individuals are characterized by an optimal small-world organization [16, 17].

Furthermore, a description of the effective resting-state networks during three different ages (children, mid-aged, and elderly) was proposed by Zhu et al. [12]: in this study, effective connectivity was estimated starting from EEG signals, but the aging effects in the effective resting-state networks were investigated only in terms of hemispheres asymmetry.

The aim of the present study was to exploit a combination of advanced methods for the estimation of cortical connectivity, the use of directed and weighted graph theoretical indices, and a classification approach to investigate differences in resting-state networks related to age.

Partial Directed Coherence (PDC, [18]) is a powerful estimator of effective connectivity: it returns information about the existence, the intensity, and the direction of the causal relations between EEG signals. In order to exploit all the information returned by the PDC, in this study, we computed the directed and weighted version of state-of-the-art graph theory indices. We extracted salient indices synthesizing the architecture of the connectivity networks elicited during the rest condition in a group of 71 healthy adults, of age ranging from 20 to 63 years. A first investigation was conducted by mean of correlation study in order to find the topological properties that are related to age. A second step focused on the characterization of two subgroups (young and middle-aged adults) and aimed to identify indices showing statistical differences between the two groups, to be used as features for classifying resting-state patterns in relation to the age of the subject.

## 2. Methods

### 2.1. Experimental Design

71 healthy subjects took part in the study (age ranging from 20 to 63 years). EEG signals were collected from 61 positions, assembled on an electrode cap (according to an extension of the 10–20 International System) during one minute of eyes-closed resting-state condition. We avoided scalp positioned reference electrodes, to minimize the effect on the signal phases. Subjects were instructed to relax and not to think of anything. After band-pass filtering (1–45 Hz + 50 Hz Notch filter), muscular and environmental artifacts were removed using a semiautomatic procedure, based on the definition of a voltage threshold (±80 *μ*V), and Independent Component Analysis [19] was applied to remove ocular artifacts related to involuntary twitch during eyes-closed condition. Particular attention was put in avoiding any preprocessing procedure that may induce changes in the phase relations between channels. EEG traces were then segmented in 1 s-epochs and the brain connectivity was estimated by means of PDC: the obtained patterns were averaged over frequency range of 1–30 Hz. The frequency band was selected in accordance with previous studies [12, 20].

### 2.2. Multivariate Autoregressive Modeling of Brain Signals

Suppose that the following multivariate autoregressive (MVAR) model is an adequate description of the dataset *Y*:

(1) $$\begin{array}{c} \overset{p}{\underset{k=0}{\sum }}\Lambda \left(\;k\;\right)Y\left(\;t-k\;\right)=E\left(\;t\;\right), \end{array}$$

where *Y*(*t*) is the data vector in time, *E*(*t*) is a vector of multivariate zero-mean uncorrelated white noise processes, Λ(*k*) is the matrix of model coefficients at lag *k*, and *p* is the model order that can be chosen by means of the Akaike Information Criteria (AIC) for MVAR processes [21]. For the dataset used in this study, the resulting optimal model order was around 20 for each subject. We checked that the amount of data points was at least an order of magnitude higher than the number of parameters to be estimated for the model. In order to investigate the spectral properties of the examined process, (1) is transformed to the frequency domain:

(2) ΛfYf=Ef,Λf=∑k=0pΛke−j2πfΔtk,

where Δ*t* is the temporal interval between two samples.

An adaptive formulation for MVAR model (AMVAR) is used in the study [22]. The time dependent parameter matrices can be estimated by means of GLKF method (described in the following).

### 2.3. Partial Directed Coherence

The PDC [18] is a full multivariate spectral measure, used to determine the directed influences between any given pair of signals in a multivariate dataset. This estimator was demonstrated to be a frequency version of the concept of Granger causality [23]. In this study, we adopted the following formulation of PDC:

(3) $$\begin{array}{c} \pi_{ij}\left(\;f\;\right)=\frac{\Lambda_{ij}\left(f\right)}{\sqrt{\sum_{k=1}^{N}\Lambda_{ik}\left(f\right)\Lambda_{ik}\left(f\right)}}, \end{array}$$

where Λ(*f*) is the matrix containing the coefficients of associated MVAR model and

(4) $$\begin{array}{c} \overset{N}{\underset{n=1}{\sum }}{\left|\;\pi_{in}\left(\;f\;\right)\;\right|}^{2}=1. \end{array}$$

Due to the normalization reported in (4), PDC values are in the interval $\begin{array}{cc} {}[0 & 1] \end{array}$. With respect to the original definition of PDC [18], according to which the estimator is normalized according to the amount of connectivity emitted from the “source” channel, here, we normalized the estimator according to the total connectivity incoming to the “target” channel (row-wise normalization instead of column-wise one), to avoid the fact that PDC from given electrode is decreased when multiple signals are emitted from it.

The formulation of the estimator proposed in [18] is based on the hypothesis of stationarity of signals included in the estimation process. Unfortunately, such hypothesis leads to a complete loss of the information about the temporal evolution of estimated information flows. For overcoming this limitation, a time-varying adaptation of PDC was introduced. The adaptation consisted of modifying the original formulation of PDC by including dependence from the time in the MVAR coefficients. Thus, the adaptive PDC estimator can be defined as follows:

(5) $$\begin{array}{c} \pi_{ij}\left(\;f,t\;\right)=\frac{\Lambda_{ij}\left(f,t\right)}{\sqrt{\sum_{k=1}^{N}\Lambda_{ki}\left(f,t\right)\Lambda_{ki}\left(f,t\right)}}, \end{array}$$

where *t* refers to a dependence of the MVAR coefficients from time and Λ_*ij*_(*f*, *t*) represents the *ij* entry of the matrix of MVAR model coefficients Λ at frequency *f* and time *t*.

In this study, we used the squared formulation of PDC due to its higher accuracy and stability [24].

### 2.4. General Linear Kalman Filter

The coefficients of the AMVAR model were estimated by means of General Linear Kalman Filter (GLKF). In the GLKF, an adaptation of the Kalman Filter to the case of multitrial time series is provided [25]. In particular, the equations at the basis of the algorithm are

(6) Qn=Gn−1Qn−1+Vn,On=HnQn+Wn,

where *O*_*n*_ represents the observation, *Q*_*n*_ is the state process, *H*_*n*_ and *G*_*n*_ are the transition matrices, and *V*_*n*_ and *W*_*n*_ are the additive noises. To obtain the connection with the time-varying MVAR, it is necessary to make the following associations:

(7) Qn=Λ1nT⋮ΛpnT,On=y11n⋯yM1n⋮⋱⋮y1Tn⋯yMTn=Yn,Gn−1=Idp,Hn=On−1,…,On−p,

where *T* denotes the number of trials, whereas *M* is the dimension of the measured process. The details of the algorithm are provided in [25]. The quality of estimation is related to the definition of two adaptation parameters, *c*1 and *c*2, which regulate the compromise between the quality of estimation and the speed of adaptation to transitions. This algorithm was developed for time-varying connectivity estimation but it can be also applied in the stationary case, to provide high stability and accuracy with multichannel data [25]. In this case, the appropriate choice of the adaptation constants allows for strengthening the estimator accuracy (*c*1 = *c*2 = 0.001).

### 2.5. Support Vector Machine (SVM)

The Support Vector Machine (SVM) was first proposed by Vapnik and has since attracted a high degree of interest in the machine learning research community [26]. SVMs are supervised learning models used for classification and regression analysis. In order to perform a binary classification (two separate classes), this method needs training data, marked as belonging to one of two categories, for introducing a separating hyperplane: this hyperplane must maximize the margin between the two classes and it is known as the optimum separating hyperplane. The details of the method are provided in [27].

### 2.6. Graph Theory Approach

A graph is an abstract representation of a network that consists of a set of vertices (or nodes) linked by means of edges (or connections) indicating the presence of some sort of interaction between the vertices. The adjacency matrix *A* is the mathematical representation of a graph. It contains the information about the connectivity structure of the graph: the entry *a*_*ij*_ is different from 0 if there is an effective link between nodes *i* and *j* and equal to 0 if no link exists.

Several indices based on the elements of such matrix can be computed for the characterization of the main properties of brain networks [28]. In this study, we used weighted versions of the indices, to take into account strength and direction of the connectivity links returned by PDC.

#### 2.6.1. Node Strength

The strength index *s* represents the total intensity associated with arcs that involve the node *i*, both ingoing ones (*in-strength*, *s*_in_) and outgoing ones (*out-strength*, *s*_out_):

(8) $$\begin{array}{c} s\left(\;i\;\right)=s_{\text{in}}\left(\;i\;\right)+s_{\text{out}}\left(\;i\;\right)=\sum_{j=V,j\neq i}w_{ji}+\sum_{j=V,j\neq i}w_{ij}, \end{array}$$

where *w*_*ij*_ is the intensity of the link from node *j* to node *i*.

#### 2.6.2. Characteristic Path Length

The characteristic path length is the average shortest path length in the network [29]. Its weighted and directed version can be defined as follows:

(9) Lw=1n∑i∈NLi=1n∑i∈N∑j∈N,j≠idijwn−1,dijw=∑auv∈gi→jwfwuv,

where *L*_*i*_ is the average distance between node *i* and all other nodes, *d*_*ij*_ is the distance between node *i* and node *j*, *f* is a map (i.e., the inverse) from weight to length, and *g*_*i*→*j*_^*w*^ is the directed shortest path from *i* to *j* (the superscript “*w*” indicates that the equation is associated with the weighted version).

#### 2.6.3. Clustering Coefficient

The clustering coefficient describes the intensity of interconnections between the neighbors of a node [29]. It is defined as the fraction of triangles around a node or the fraction of node's neighbors that are neighbors of each other. The weighted directed version of clustering coefficient is defined as follows:

(10) Cw=1n∑i∈Ntiwkiout+kiinkiout+kiin−1−2∑j∈Nwijwji,

where *t*_*i*_ represents the number of triangles involving node *i*, *k*_*i*_^in^ and *k*_*i*_^out^ are the number of incoming and outcoming edges of nodes *i*, respectively, and *w*_*ij*_ is the entry of connectivity matrix (i.e., weight of connection from node *j* to node *i*).

#### 2.6.4. Global Efficiency

The global efficiency is the average of the inverse of the geodesic length (shortest path between two nodes in the network) and represents the efficiency of the communication between all the nodes in the network [30]. The weighted and directed formulation is shown in the following equation:

(11) $$\begin{array}{c} {\text{GlobEff}}^{w}=\frac{1}{n}\sum_{i\in N}\frac{\sum_{j\in N,j\neq i}{\left(d_{ij}^{w}\right)}^{-1}}{n-1}, \end{array}$$

where *d*_*ij*_^*w*^ is the weighted distance between node *i* and node *j*.

#### 2.6.5. Local Efficiency

The local efficiency is the average of the global efficiencies computed on each subgraph belonging to the network and represents the efficiency of the communication between all the nodes around the node *i* in the network [30]. Its weighted and directed version is defined as follows:

(12) $$\begin{array}{c} {\text{LocEff}}^{w}=\frac{1}{2n}\sum_{i\in N}\frac{\sum_{j,h\in N,j\neq i}\left(w_{ij}+w_{ji}\right)\left(w_{ih}+w_{hi}\right)\left({\left[d_{jh}^{w}\left(N_{i}\right)\right]}^{-1}+{\left[d_{hj}^{w}\left(N_{i}\right)\right]}^{-1}\right)}{\left(k_{i}^{\text{out}}+k_{i}^{\text{in}}\right)\left(k_{i}^{\text{out}}+k_{i}^{\text{in}}-1\right)-2\sum_{j\in N}w_{ij}w_{ji}}, \end{array}$$

where *w*_*ij*_ is the entry of connectivity matrix (i.e., weight of connection from node *j* to node *i*), *k* ^in^ and *k* ^out^ are the number of incoming and outcoming edges of node *i*, respectively, and *d*_*ij*_^*w*^ is the weighted distance between node *i* and node *j*.

#### 2.6.6. Weight

The weight is the mean value of all connections in the filtered network:

(13) $$\begin{array}{c} \text{Weight}=\frac{\sum_{j,i\in N}w_{ji}}{L}, \end{array}$$

where *w*_*ji*_ is the entry of weighted connectivity matrix and *L* is the number of filtered connections.

### 2.7. Statistical Analysis

A weighted directed adjacency matrix was extracted for each subject by applying a threshold able to maintain the 20% of the stronger connections. As a first step of analysis, a correlation study (Spearman correlation) was performed between age and each global index (all the indices described in the previous section except for the local index* node strength*) for the whole population (71 healthy subjects). Then, two subgroups were selected for classification analysis with respect to age: “young” group (20 subjects with age: 23.8 ± 1.05 years) and “middle-aged” group (20 subjects with age: 46.05 ± 5.27 years). To characterize the resting-state connectivity patterns elicited by the two age groups, a first investigation was performed by means of the local index. The node strength was computed for each subject and a Grand Average was performed for each group in order to identify the brain regions mainly involved in the resting-state network. Furthermore, to quantify the differences related to the network architecture of the two groups of subjects, the global graph indices described above were subjected to the following steps:

(1) Statistical comparison (two-sample Student's *t*-test) for a significance level of 5% between indices from young and middle-aged subjects networks.
(2) Classification of the extracted features by means of SVM classifier [26] (linear kernel).

Before computing the first step, the hypothesis of normal distribution was verified applying the Kolmogorov-Smirnov one-sample test. *t*-tests were performed for investigating which indices were significantly different between the populations: this step was important to allow for choosing the features to be used in the classification process. For the second step, a Leave One Out approach has been implemented to perform the classification. In particular, graph indices extracted from a single subject were used for classifying him/her in one of the two groups, using the indexes achieved by other randomly chosen 30 subjects (15 young and 15 middle-aged adults) as training data for SVM classifier. Each subject was tested singularly for 50 times and each iteration was characterized by a different combination of the 30 subjects used for the classifier training. During each iteration, the algorithm returned score equal to 1 for right classification and 0 otherwise. The classification performance was obtained for each subject among the iterations; then, total, young, and middle-aged performances were computed by performing the average of the performances among all the subjects belonging to each group, respectively.

## 3. Results

**Figure 1:**
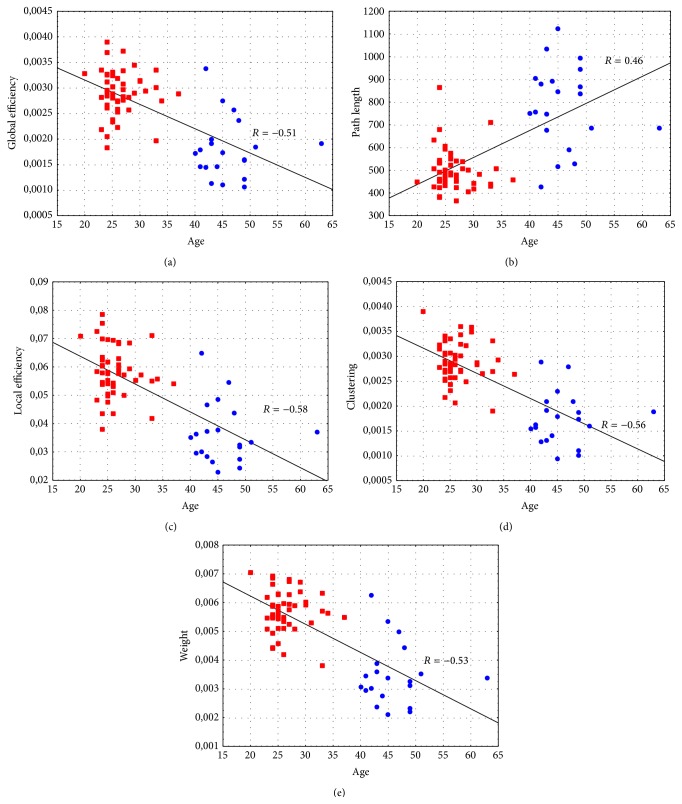
Results of the correlation analysis: (a) Global Efficiency-Age (Spearman's correlation coefficient *R* = −0.51, *p* < 10^−3^), (b) Path Length-Age (Spearman's correlation coefficient *R* = 0.46, *p* < 10^−3^), (c) Local Efficiency-Age (Spearman's correlation coefficient *R* = −0.58, *p* < 10^−3^), (d) Clustering-Age (Spearman's correlation coefficient *R* = −0.56, *p* < 10^−3^), and (e) Weight-Age (Spearman's correlation coefficient *R* = −0.53, *p* < 10^−3^). Red squares denote subjects with age ranging from 20 to 40 years, whereas blue circles denote subjects with age ranging from 41 to 65 years.

All the investigated indices correlate with age: in particular, Spearman's correlation coefficient *R* is negative in all cases except for the path length (Figure 1) indicating that in the effective resting-state networks the communication (measured by means of* efficiencies*,* path length,* and* clustering*) and the global strength (measured by means of* weight*) tend to decrease with the age.

**Figure 2:**
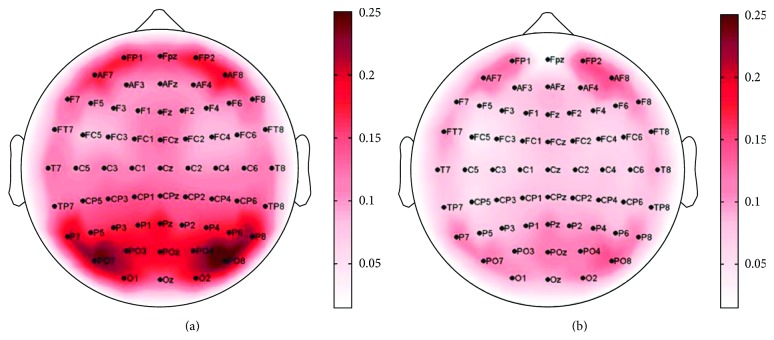
Grand Average of the node strength index: (a) young group, (b) middle-aged group. The scalp is seen from the above, with the nose pointing to the upper part of the page. Color bars code for strength value.

After the preliminary investigation about the network properties in relation to the age, we focused on the characterization of effective resting-state networks related to two age subgroups (young and middle-aged). In Figure 2, the Grand Average of the node strength index is shown on a scalp map for each group.  Figure 2(a) shows the strength map for the young individuals: the obtained pattern reveals a symmetric behavior with respect to the hemispheres and the parieto-occipital areas are mainly involved in the resting-state network (regions characterized by stronger connections), with weaker sources in frontal region. Instead, Figure 2(b) shows the strength Grand Average obtained for middle-aged subjects: also, in this case, the frontal and parieto-occipital areas characterize the pattern. However, this analysis reveals that the strength values obtained for the young group are higher than those obtained for the middle-aged group and that these latter are more balanced for the frontal and parieto-occipital areas.

**Figure 3:**
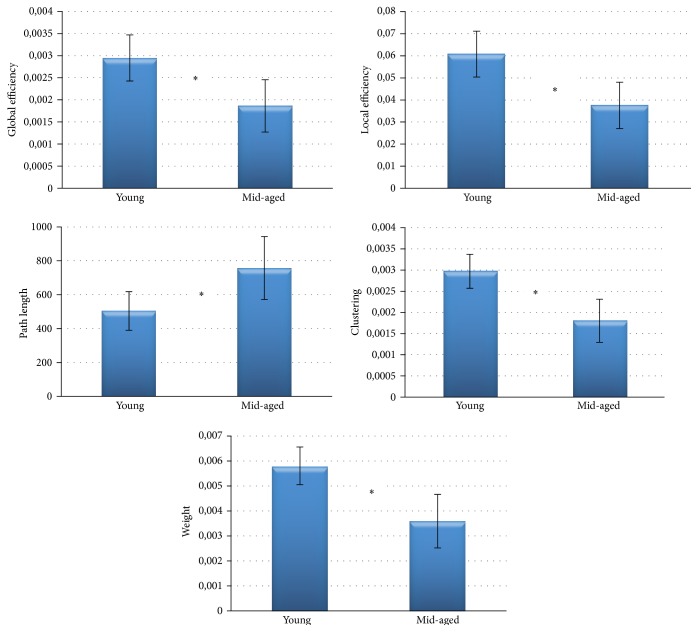
Bar diagrams reporting indices mean value achieved for the two age-related groups. Two-sample *t*-test for a significance level of 5% was performed between the two populations: the symbol (*∗*) highlights a significant difference between the two groups.

The global indices extracted separately from the two subgroups were subjected to an independent *t*-test for a significance level of 5%. Bar diagrams reported in Figure 3 show mean values of the indices achieved. In accordance with the correlation results, the statistical comparison reveals a significantly higher value of path length and significantly lower values of the other indices in the middle-aged group when compared to the young group.

**Figure 4:**
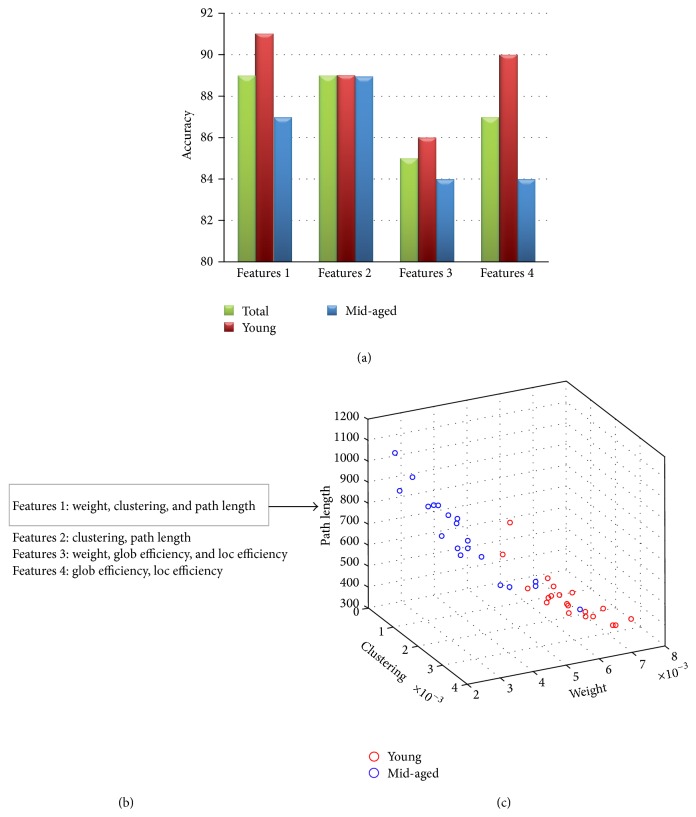
(a) Bar diagrams reporting classification accuracy achieved for the total group (40 subjects) and separately for the two age-related groups (red for 20 young subjects, blue for 20 middle-aged adults). (b) Groups of indices used as features for classification analysis. (c) 3D scatter plot for Features 1: red circles denote young subjects, whereas blue circles denote middle-aged subjects.

At last, to validate at the single subject level the obtained results, four different groups of indices were used as features in the classification analysis (Figure 4(b)). Classification performances reported in Figure 4(a) reveal accuracies higher than 82% for all the examined cases.

## 4. Discussion

Studying the resting-state activity and connectivity has gained more and more importance in the last years, as a consequence of the definition of the so-called Default Mode Network in functional MRI studies and, more importantly, of its alterations in many different pathologies. However, its neuroelectrical counterpart, though intensively studied, has not provided stable and repeatable results. One of the most difficult aspects of the problem is probably the high variability intrinsic in neuroelectrical signals and their low Signal to Noise Ratio.

In this paper, we described a methodology able to combine advanced techniques for the estimation of stable, repeatable effective connectivity and graph theory for describing and classifying age-related changes in resting-state networks. The method we used for the estimation of brain networks (a modified formulation of Partial Directed Coherence, [18]) is able to return information about the existence, the weight, and the direction of the connection flow between brain regions. In the last years, different formulations of PDC have been proposed [18, 31–33] with the purpose to address specific issues related to the original PDC, like the decrease of PDC from a given channel when multiple signals are emitted from that channel, the effect of differences in amplitude (e.g., signals of different nature, like EEG and EMG, EEG, and LFP), and the interpretability of connectivity values. In this paper, as the study focuses on the variations of effective connectivity patterns in relation to the age, a renormalization of the original PDC, performed with respect to all connectivity links incoming to each channel, was applied to signals recorded by means of high density EEG during rest condition. As a general consideration, when dealing with signals with different scales (like EEG and EMG, EEG and LFP, and so on, or signals resulting from recordings on patients) or when being interested in the interpretation of the connectivity values, the use of gPDC or renormalized PDC [31] should be preferred.

The use of a General Linear Kalman Filtering approach [25] allowed for overcoming the limitation of the number of channels simultaneously included in a single AMVAR model, permitting a full multivariate approach even with a high number of nodes (61 EEG channels). The advantage of using a multivariate approach with respect to a pairwise one is significant, in terms of accuracy of the results and reduction of false positives, as shown, for instance, in [34]. Its main limitation is due to the amount of data necessary to provide a correct estimation of a high dimensional model, with a large number of parameters. This usually compels to an a priori selection of regions or channels to be studied, which is more difficult in a resting-state condition with respect to task related data. Our GLKF approach, on the contrary, has shown excellent properties of stability and accuracy even with a high number of channels (up to 100) and an amount of data reduced to few minutes of recordings [24, 34].

The plethora of information returned by the connectivity estimation and the complexity of the resulting networks requires pointing to a graph theoretical approach to extract and quantify the relevant information about the network organization. An important aspect of this study is that we used the directed and weighted formulation of main graph indices, to exploit the richness of information provided by the connectivity estimation.

All these methodological steps proved to be a valid procedure for the description of age-related changes in human brain at rest. In fact, results of correlation study for the whole group of 71 subjects indicated that the organization of brain networks turns to a more random (less structured) condition with the normal aging. A decrease of connections weight, efficiencies, and clustering and an increase of the characteristic path length denote that “middle-aged” networks are less organized and characterized by lower power with respect to young age.

The selection of two subgroups of subjects in accordance with their “young” or “middle” age has revealed differences in the resting-state EEG pattern. The use of the local node strength index allowed for identifying the brain regions mainly involved in the eyes-closed resting-state network. Indeed, in both groups, a prevalent role of frontal and parieto-occipital areas characterizes the obtained patterns. However, results showed a weakening of connections strength with the aging (in agreement with the correlation between the weight index and age): this behavior is more related to the parieto-occipital area.

Furthermore, significant differences were found by means of global network indices. This result is important not only in the context of the study of normal ageing, but also in the study of pathological alterations of the brain intrinsic organization. In fact, such alterations are often expressed in terms of their distance from a physiological conditions as represented by healthy subjects. The use of normative databases of healthy subjects is then desirable and it is important to understand how such normative parameters have to be computed and how homogeneous the population has to be to provide a correct baseline.

Graph indices studied in this work proved to be good descriptors for the architecture of human brain networks. Their use of a combination of such indices as a vector of features for the classification analysis allowed for obtaining high performances: in fact, in all the cases examined, the accuracy is higher than 82%.

Results of this study show that the proposed approach allows for evaluating age-related differences in neuroelectrical resting-state activity and for classifying these differences at single subject level just on the basis of 1-minute recordings of eyes-closed resting condition. As for the physiological conclusion, the results of this study suggest that the transition from young to middle age impacts the topological configuration of resting-state networks towards a decrease of the small-world configuration and to a tendency to the random configuration (decrease of clustering and increase of path length). This is in accordance with the literature [3, 10, 11, 14], adding, at the same time, more details about the nature of such changes (as conveyed by the weighted, directed indices) and a stronger reliability of the data.

Future works will focus on an enlargement of the subjects sample (including the 65–85 years' range) to provide a baseline for pathologies typically involving elderly age. The suggested approach can be easily extended to more specific topological properties of the resting-state network, in order to answer questions related to the involvement of specific circuits or regions of interest in view of the application to specific clinical conditions, thus providing a versatile tool for the study of the brain intrinsic organization starting just from few minutes of noninvasive EEG recordings.
